# Month of birth, vitamin D and risk of immune-mediated disease: a case control study

**DOI:** 10.1186/1741-7015-10-69

**Published:** 2012-07-06

**Authors:** Giulio Disanto, George Chaplin, Julia M Morahan, Gavin Giovannoni, Elina Hyppönen, George C Ebers, Sreeram V Ramagopalan

**Affiliations:** 1 Wellcome Trust Centre for Human Genetics, University of Oxford, Oxford, UK OX3 7BN; 2 Department of Clinical Neurology, University of Oxford, Oxford, UK OX3 9DU; 3 Department of Anthropology, The Pennsylvania State University, Pennsylvania, United States of America, 16802; 4 Blizard Institute of Cell and Molecular Science, Queen Mary University of London, Barts and The London School of Medicine and Dentistry, London, UK, E1 2AT; 5 Centre for Paediatric Epidemiology and Biostatistics and MRC Centre of Epidemiology for Child Health, UCL Institute of Child Health, London, UK, WC1N 1EH; 6 London School of Hygiene and Tropical Medicine, London, UK, WC1E 7HT

## Abstract

**Background:**

A season of birth effect in immune-mediated diseases (ID) such as multiple sclerosis and type 1 diabetes has been consistently reported. We aimed to investigate whether season of birth influences the risk of rheumatoid arthritis, Crohn's disease, ulcerative colitis and systemic lupus erythematosus in addition to multiple sclerosis, and to explore the correlation between the risk of ID and predicted ultraviolet B (UVB) light exposure and vitamin D status during gestation.

**Methods:**

The monthly distribution of births of patients with ID from the UK (n = 115,172) was compared to that of the general population using the Cosinor test. Predicted UVB radiation and vitamin D status in different time windows during pregnancy were calculated for each month of birth and correlated with risk of ID using the Spearman's correlation coefficient.

**Results:**

The distributions of ID births significantly differed from that of the general population (*P *= 5e^-12^) with a peak in April (odds ratio = 1.045, 95% confidence interval = 1.024, 1.067, *P *< 0.0001) and a trough in October (odds ratio = 0.945, 95% confidence interval = 0.925, 0.966, *P *< 0.0001). Stratification by disease subtype showed seasonality in all ID but Crohn's disease. The risk of ID was inversely correlated with predicted second trimester UVB exposure (Spearman's rho = -0.49, *P *= 0.00005) and third trimester vitamin D status (Spearman's rho = -0.44, *P *= 0.0003).

**Conclusions:**

The risk of different ID in the UK is significantly influenced by the season of birth, suggesting the presence of a shared seasonal risk factor or factors predisposing to ID. Gestational UVB and vitamin D exposure may be implicated in the aetiology of ID.

## Background

Complex disorders such as immune-mediated diseases (ID) are defined as conditions that have no single cause but result from a combination of genetic and environmental factors and their interactions. ID affect approximately 5% to 10% of the developed world and the overall incidence seems to be increasing [1]. This observation suggests that changes in environment and lifestyle play a central role in influencing prevalence.

Seasonality dominates the global environment and diet is closely related to seasonality by the effect of these environmental fluctuations on agriculture [2]. Seasonal factors can potentially act even before birth, when, according to the 'fetal origin of adult disease hypothesis', environmental influences leading to changes in embryonic or fetal tissue structure and function can influence the risk of adult physiological and pathological conditions [3,4]. As a consequence, being born at a certain time of the year may influence susceptibility to disease later in life. Indeed, month of birth effects have already been documented in ID such as multiple sclerosis (MS) and type 1 diabetes (T1D) [5-7]. In addition to MS and T1D, a few other studies have investigated the presence of a month of birth effect in other ID. However, poor sample sizes and inadequate statistical methods have significantly hampered these attempts and results are inconsistent [8-16].

The mechanisms involved in the pathogenesis of ID are variable, and both adaptive and innate immune responses have been implicated in diseases such as MS, rheumatoid arthritis (RA), systemic lupus erythematosus (SLE), Crohn's disease (CD) and ulcerative colitis (UC) [17-20]. For example, in MS and RA tolerance breakdown is thought to cause immune-mediated demyelination of the central nervous system and cartilage and bone destruction respectively [18,21]. By contrast, several lines of evidence suggest that CD and UC arise from an inappropriate immune reaction to the intestinal microbiota in genetically predisposed hosts [20]. Despite these differences, an abnormal activation of the immune system is a common thread linking these conditions and several observations indicate that similar genetic pathways and environmental agents, such as vitamin D deficiency, smoking behaviour and various infections, are involved in the pathogenesis of these disorders [18-20,22-25].

This led us to the *a priori *hypothesis that a similar seasonality of birth may be present among different ID. We investigated whether the month of birth influences susceptibility to RA, SLE, CD and UC in addition to MS using the largest cohort to date to investigate these effects (n = 115,172). Since all these conditions have been linked to vitamin D deficiency [23,24], we also tested whether the risk of disease by month of birth follows the same seasonal distribution of predicted ultraviolet B (UVB) light radiation and 25-hydroxyvitamin D (25-OH-D) levels during gestation.

## Methods

Month of birth for MS (n = 15,492), RA (n = 39,666), SLE (n = 4,046), CD (n = 20,574) and UC (n = 23,892) patients seen by a doctor between 1997 and 2009 in Scotland and between 2003 and 2009 in England were obtained from the National Health Service (NHS) Scotland and the English Hospital Episode Statistics (HES). For MS, an additional cohort of patients (n = 11,502) and matched controls was collected as previously described [5], giving a total of 26,994 MS patients. General population controls were obtained from the General Register Office http://www.gro-scotland.gov.uk/ and the Office for National Statistics http://www.ons.gov.uk/. Scottish controls were based on month of birth registration between 1954 and 1973 and actual month of birth between 1974 and 1990. English controls were based on actual month of birth between 1950 and 1990. In total, month of birth data were collected for 115,172 patients with ID (26,162 English and 89,010 Scottish, Table 1) as well as for 3,028,621 Scottish and 29,202,890 English controls.

**Table 1:** Total number of patients with immune-mediated diseases used in the analysis

	Multiple sclerosis	Rheumatoid arthritis	Systemic lupus erythematosus	Crohn's disease	Ulcerative colitis	All immune-mediated diseases
**England**	13,075	4,747	1,622	2,463	4,255	26,162
**Scotland**	13,919	34,919	2,424	18,111	19,637	89,010
**Total**	26,994	39,666	4,046	20,574	23,892	115,172

We compared cases and controls using the Cosinor test, which is able to capture seasonal distributions and is particularly suitable for relatively simple and symmetric seasonal patterns. This test fits a generalized linear model under the Poisson distribution using sine and cosine terms that together describe the sinusoid. In addition to statistical significance, the model provides information on the amplitude (the height) and the phase (the peak point from 1 to 12 indicating months) of the predicted sinusoid [26]. Monthly odds ratios (OR) were also calculated by comparing frequencies of patients and controls born in a certain month versus the rest of the year.

Average monthly UVB radiation at the wavelength of 305 nm at noon (joules/square metre) in England and Scotland between 1979 and 1992 was obtained from the National Aeronautics and Space Administration's Total Ozone Mapping Program on the Nimbus 7 satellite, as previously described [27]. Average monthly 25-OH-D levels were collected from a large cohort of adult Scottish and English women (n = 3,787) as previously described [28] and used as a proxy for the seasonal variation in gestational vitamin D status. Average predicted UVB exposure as well as vitamin D status during the first, second and third trimesters of gestation were calculated for each month of birth and tested for correlation with risk of ID (monthly OR) using the Spearman's correlation coefficient. Statistical analyses were performed using R.

## Results

**Figure 1:**
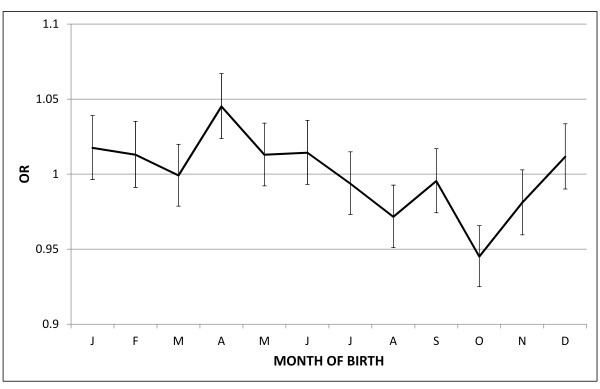
**Odds ratio distribution with 95% CI based on month of birth in all immune-mediated diseases (n = 115,172) versus general population**. April peak and October trough of risk can be observed.

To assess whether month of birth influences susceptibility to immune disorders, we initially compared the distribution of all patients with ID with that of the general population. Using the Cosinor test, the birth distribution of patients with ID was found to follow a seasonal distribution as compared with the general population (*P *= 5e^-12^, amplitude = 0.033, phase = 3.08, low point = 9.08). When monthly ORs were calculated, a statistically significant peak was found in April (OR = 1.045, 95%CI = 1.024 to 1.067, *P *< 0.0001) and a significant trough exactly six months later in October (OR = 0.945, 95%CI = 0.925 to 0.966, *P *< 0.0001). A smaller deficit was also detected in August (OR = 0.972, 95%CI = 0.951 to 0.9927, *P *= 0.008) (Figure 1). The peak to trough ratio indicated the presence of a 6.5% increased risk for individuals born in April versus those born in October (OR = 1.065, 95%CI = 1.035 to 1.096, *P *< 0.0001).

**Figure 2:**
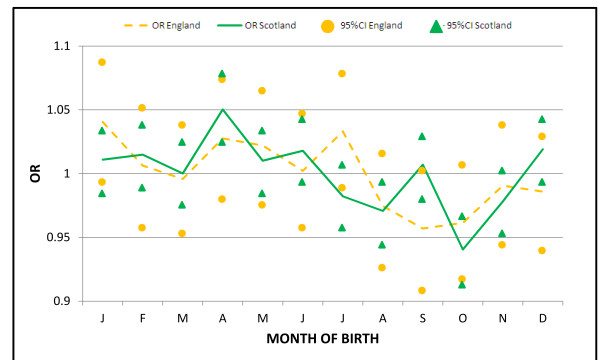
**Odds ratio distribution based on month of birth in England and Scotland**. The highest and lowest odds ratios are observed in Scotland but 95%CI substantially overlap.

When the analysis was performed according to country, the seasonal effect appeared to be present in both England and Scotland (Scotland *P *= 5e^-10^, amplitude = 0.034, phase = 3.05, low point = 9.05; England *P *= 0.005, amplitude = 0.032, phase = 3.23, low point = 9.23). The highest and lowest monthly ORs were found in the Scottish population; however, 95%CIs were substantially overlapping (Figure 2).

The seasonality of birth detected by grouping all patients with ID could arise from a single disease such as MS, for which the presence of a month of birth effect has already been described. We therefore stratified the analysis by disease type. The Cosinor test indicated the presence of clear seasonality in all ID but CD: MS, *P *= 5e^-06^; amplitude = 0.041, phase = 4.12, low point = 10.12; RA, *P *= 5e^-04^, amplitude = 0.032, phase = 2.69, low point = 8.69; UC, *P *= 5e^-04^, amplitude = 0.04, phase = 2.74, low point = 8.74; SLE, *P *= 0.025, amplitude = 0.063, phase = 2.89, low point = 8.89; CD, *P *> 0.05. When calculating monthly ORs, a peak in spring and a deficit in autumn could be observed in each ID apart from CD, in which a January rather than spring peak was found. Birth percentages and monthly ORs with 95%CIs are presented in Table 2.

**Table 2:** Birth percentages and monthly odds ratios with 95%CI for each and all immune-mediated diseases

Month	All immune-mediated diseases			Multiple sclerosis			Rheumatoid arthritis		
	Birth %	OR	95% CI	Birth %	OR	95% CI	Birth %	OR	95% CI
**Jan**	**8.63**	**1.02 +**	**0.99 to 1.04**	8.51	1.01	0.97 to 1.05	8.44	0.99	0.95 to 1.03
**Feb**	7.90	1.01	0.99 to 1.04	7.76	0.99	0.95 to 1.03	7.94	1.02	0.98 to 1.06
**Mar**	8.88	1.00	0.98 to 1.02	8.67	0.97	0.93 to 1.01	8.99	1.01	0.98 to 1.05
**Apr**	**8.77**	**1.05 +**	**1.02 to 1.07**	**8.79**	**1.05 +**	**1.002 to 1.09**	**8.78**	**1.05 +**	**1.01 to 1.08**
**May**	8.83	1.01	0.99 to 1.03	**9.41**	**1.08 +**	**1.04 to 1.13**	8.64	0.99	0.95 to 1.03
**Jun**	8.44	1.01	0.99 to 1.04	8.70	1.04	1.01 to 1.09	8.47	1.02	0.98 to 1.06
**Jul**	8.49	0.99	0.97 to 1.01	8.51	0.99	0.95 to 1.04	8.37	0.98	0.94 to 1.01
**Aug**	**8.16**	**0.97 -**	**0.95 to 0.99**	8.20	0.98	0.94 to 1.02	**8.14**	**0.97 -**	**0.93 to 1.00**
**Sep**	8.12	1.00	0.97 to 1.02	7.94	0.96	0.92 to 1.01	8.10	1.00	0.96 to 1.03
**Oct**	**8.05**	**0.95 -**	**0.92 to 0.97**	**8.08**	**0.96 -**	**0.92 to 1.00**	**8.20**	**0.96 -**	**0.93 to 0.99**
**Nov**	7.61	0.98	0.96 to 1.00	**7.43**	**0.96 -**	**0.91 to 1.00**	7.65	0.99	0.95 to 1.02
**Dec**	8.11	1.01	0.99 to 1.03	8.01	1.00	0.96 to 1.04	**8.30**	**1.04 +**	**1.00 to 1.07**

**Month**	**Ulcerative colitis**			**Systemic lupus erythematosus**			**Crohn's disease**		
	**Birth %**	**OR**	**95% CI**	**Birth %**	**OR**	**95% CI**	**Birth %**	**OR**	**95% CI**

**Jan**	8.63	1.02	0.97 to 1.06	**9.57**	**1.14 +**	**1.03 to 1.27**	**8.99**	**1.06 +**	**1.01 to 1.11**
**Feb**	**8.07**	**1.04 +**	**0.99 to 1.09**	7.86	1.00	0.90 to 1.13	7.84	1.01	0.96 to 1.06
**Mar**	8.90	1.00	0.96 to 1.05	8.75	0.98	0.88 to 1.09	8.94	1.01	0.96 to 1.06
**Apr**	**8.92**	**1.06 +**	**1.02 to 1.11**	8.85	1.05	0.95 to 1.18	8.54	1.02	0.97 to 1.07
**May**	8.73	1.00	0.96 to 1.05	**9.71**	**1.12 +**	**1.01 to 1.24**	**8.40**	**0.96 -**	**0.91 to 1.01**
**Jun**	8.25	0.99	0.94 to 1.04	7.61	0.90	0.81 to 1.02	8.44	1.02	0.97 to 1.07
**Jul**	8.44	0.99	0.94 to 1.03	8.58	1.00	0.90 to 1.12	8.75	1.03	0.98 to 1.08
**Aug**	**8.15**	**0.97 -**	**0.93 to 1.02**	8.40	1.00	0.90 to 1.12	8.13	0.97	0.92 to 1.02
**Sep**	8.18	1.00	0.96 to 1.05	**7.51**	**0.91 -**	**0.81 to 1.02**	**8.45**	**1.04 +**	**0.99 to 1.10**
**Oct**	**7.91**	**0.93 -**	**0.88 to 0.97**	7.93	0.94	0.84 to 1.05	**7.89**	**0.92 -**	**0.87 to 0.97**
**Nov**	7.69	0.99	0.95 to 1.04	**7.09**	**0.91 -**	**0.81 to 1.03**	7.78	1.00	0.95 to 1.06
**Dec**	8.13	1.01	0.97 to 1.06	8.13	1.02	0.91 to 1.14	7.84	0.97	0.93 to 1.03

**+ and - indicate highest and lowest odds ratios, respectively. CI: confidence intervals; OR: odds ratio**.

**Figure 3:**
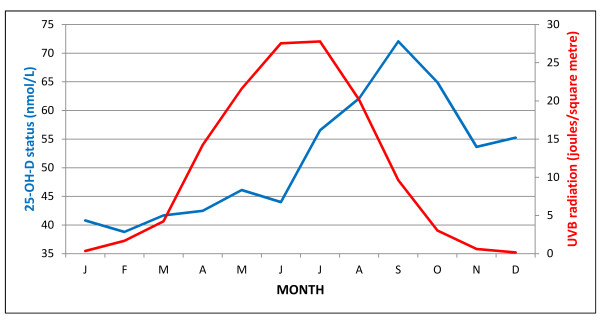
**Correlation between monthly ultraviolet B radiation from the NASA's Total Ozone Mapping Program and 25-hydroxyvitamin D levels from the general UK population**. The seasonal distribution of 25-hydroxyvitamin D levels is shifted approximately two to three months later than that of ultraviolet B radiation.

We next investigated whether the monthly risk of ID inversely correlated with predicted gestational UVB exposure and vitamin D status during different trimesters of pregnancy. Based on the Nimbus 7 satellite, UVB radiation in the UK reaches the minimum and maximum levels during winter (December to January) and summer (June to July), respectively. The highest and lowest 25-OH-D levels were collected during September and February respectively [28]. Figure 3 shows the direct relation between UVB radiation and vitamin D status and the amount of time required for a change in UVB to impact on vitamin D metabolism. The peak and the trough of 25-OH-D levels are shifted approximately two to three months later than UVB radiation (two months lag: Spearman's rho = 0.91, *P *< 2.2e^-16^; three months lag: Spearman's rho = 0.88, *P *= 0.002). This is consistent with previous reports [29].

**Figure 4:**
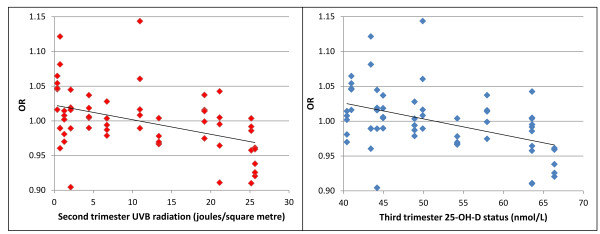
**Inverse correlation between risk of immune-mediated diseases and predicted second trimester ultraviolet B exposure (left panel) and third trimester vitamin D status (right panel)**.

We found that the monthly risk of ID inversely correlated with predicted UVB exposure during the second trimester of pregnancy (Spearman's rho = -0.49, *P *= 0.00005). Similarly, predicted maternal 25-OH-D levels were also inversely associated with risk of ID but the negative correlation was shifted to the third trimester (Spearman's rho = -0.44, *P *= 0.0003) (Figure 4).

## Discussion

We report here the largest study performed on ID and seasonality of birth. When patients with different conditions were grouped together, a clear seasonal birth distribution was observed with a peak in April and a trough exactly six months later in October. The effect size of being born at the 'wrong time' appears very low, with the highest ORs being under 1.1. However, considering the increased risk of all ID in the rest of the year versus October-born individuals and the proportion of the population born in months other than October, the population proportional attributable risk per cent is 5.05%. This suggests that approximately 5% of ID cases could be prevented by ameliorating the risk factor responsible for the seasonal distribution of ID births. The season of birth effect was particularly clear in Scotland as compared with England, but no prominent differences between the two sites could be observed.

That the risk of MS varies by month of birth has already been shown in a number of regions, including Canada, Denmark, Sweden, Sardinia, Finland, England, Scotland and Australia [5,30-34]. We further confirmed these findings by increasing the sample size of a previously analysed cohort of UK patients with MS [5]. Based on the Cosinor test, RA, UC and SLE births also followed a clear seasonal distribution. Notably, all the predicted sinusoids peaked around the same period, with phases ranging from 2.69 to 4.12 (late winter-spring). In contrast to other ID, the distribution of CD births was not seasonal.

The presence of seasonality of births among patients with UC but not CD is interesting but difficult to interpret. Somehow similar is the observation that the season of birth effect in MS is present among patients with relapsing remitting but not primary progressive MS [35]. It is therefore plausible to observe such differences between similar but distinct phenotypes. Furthermore, increasing evidence supports the presence of gene-environment interactions in disease aetiology [36,37] and particular genetic variants could be involved and mediating the season of birth effect. Although many genetic variants influence the risk of both UC and CD, many others (including variants located within the major histocompatibility complex) appear to be disease specific and this could contribute to the observed difference between UC and CD births [20,38-41].

A recent Australian study reported an inverse association between the risk of MS and UVB exposure during the first trimester of gestation [31]. However, the sample size was relatively small (n = 1,524) and thus analysis had to be performed using bi-monthly periods. Furthermore, the seasonal variation of 25-OH-D levels was not investigated and no other studies have tried to answer the same question in ID other than MS. We found that the risk of ID was inversely associated with predicted second trimester UVB exposure and third trimester vitamin D status. These findings are interesting since several lines of evidence now support a role for vitamin D deficiency in the pathogenesis of ID [23,24]. Notably, vitamin D production is strictly dependent on UVB radiation and vitamin D levels therefore follow a seasonal distribution [23]. This is also the case among pregnant women, whose vitamin D status largely depends on season and follows the same distribution as the levels of the general population [28,42,43]. Furthermore, in utero vitamin D deficiency has a significant effect on the developing immune system and our group has recently shown that genes associated with MS, RA, CD, SLE and T1D are significantly enriched for vitamin D receptor binding sites [44-46]. In addition to its well-known immunological roles, this exceptionally pleiotropic hormone has been implicated in autophagy and mucosal barrier homeostasis, which are thought to play a pathogenic role in CD and UC [20,47,48]. It may be that in utero vitamin D deficiency, in conjunction with individual genetic variation and subsequent exposure to other environmental agents, may then lead to disease specificity. Notably, schizophrenia is also influenced by the season of birth and a recent study has shown that neonatal vitamin D levels are significantly associated with risk of schizophrenia later in life [49,50]. Future studies should try to answer the same question in MS as well as in other ID.

This study has limitations. Information on sex and ethnicity was not available and this may have confounded our results. Furthermore, the data we gathered from the Scottish NHS and the English HES could not be restricted to UK born but only to UK resident individuals. However, the enormous sample size (115,172 ID cases), the relatively homogeneous Scottish population and the strong *a priori *evidence for a month of birth effect in MS make the risk of a spurious association improbable. Furthermore, it is striking that the ID analysed (apart from CD) show a similar seasonal risk distribution, which is also the one reported in patients with T1D [6,7]. This makes the data unlikely to be a chance finding.

We were limited to using average UVB radiation and general population vitamin D measures, which may differ from the individual maternal exposures. It is important to note that our UVB and vitamin D correlation analysis does not prove causation and that, although the vitamin D hypothesis is supported by both epidemiological and functional observations, seasonality dominates many features of the global environment and other seasonal factors may play a role in determining the risk of ID. Climate, temperature, infectious disease and maternal nutrition are all characterised by seasonality and thus represent excellent candidate factors.

## Conclusions

The susceptibility to different ID in the UK is influenced by the season of birth. This is particularly clear in patients with MS, RA, UC and SLE and suggests that at least some proportion of ID risk is preventable. Gestational vitamin D deficiency appears to be a plausible causative agent. The identification of the seasonal factor or factors responsible for such observations will be crucial for disease prevention strategies.

## Abbreviations

CD: Crohn's disease; HES: Hospital Episode Statistics; ID: immune-mediated disease; MS: multiple sclerosis; NHS: National Health Service; OR: odds ratio; RA: rheumatoid arthritis; SLE: systemic lupus erythematosus; T1D: type 1 diabetes; UC: ulcerative colitis; UVB: ultraviolet B; 25-OH-D: 25-hydroxyvitamin D.

## Competing interests

The authors declare that GD is funded by a research fellowship FISM-Fondazione Italiana Sclerosi Multipla-Cod.: 2010/B/5. JMM is funded by the MS Society of Australia and the UK. GG serves on scientific advisory boards for Merck Serono and Biogen Idec and Vertex Pharmaceuticals; served on the editorial board of *Multiple Sclerosis*; has received speaker honoraria from Bayer Schering Pharma, Merck Serono, Biogen Idec, Pfizer Inc., Teva Pharmaceutical Industries Ltd.-sanofiaventis, Vertex Pharmaceuticals, Genzyme Corporation, Ironwood, and Novartis; has served as a consultant for Bayer Schering Pharma, Biogen Idec, GlaxoSmithKline, Merck Serono, Protein Discovery Laboratories, Teva Pharmaceutical Industries Ltd.-sanofiaventis, UCB, Vertex Pharmaceuticals, GW Pharma, Novartis, and FivePrime; serves on the speakers bureau for Merck Serono; and has received research support from Bayer Schering Pharma, Biogen Idec, Merck Serono, Novartis, UCB, Merz Pharmaceuticals, LLC, Teva Pharmaceutical Industries Ltd-sanofiaventis, GW Pharma, and Ironwood. EH holds a Department of Health (UK) Public Health Career Scientist Award. The Centre for Paediatric Epidemiology and Biostatistics, benefits from funding support from the MRC in its capacity as the MRC Centre of Epidemiology for Child Health. Research at the University College London Institute of Child Health and Great Ormond Street Hospital for Children NHS Trust benefits from R&D funding received from the NHS Executive. GCE serves on the editorial boards of the *International Multiple Sclerosis Journal *and *Multiple Sclerosis *and as Section Editor for *BMC Medical Genetics*; has received funding for travel or speaker honoraria from Bayer Schering Pharma, sanofiaventis, Roche, and UCB; has served as a consultant to Biopartners, Bayer Schering Pharma, Howrey LLP, Heron Health, and Eli Lilly and Company; and receives research support from Bayer Schering Pharma, the Multiple Sclerosis Society of the United Kingdom, and the Multiple Sclerosis Society of Canada Scientific Research Foundation. SVR receives research support from the Multiple Sclerosis Society of Canada Scientific Research Foundation and the Multiple Sclerosis Society of the United Kingdom. GC reports no competing interests. All authors have no relationships with companies that might have an interest in the submitted work in the previous three years; their spouses, partners, or children have no financial relationships that may be relevant to the submitted work; and have no non-financial interests that may be relevant to the submitted work.

## Authors' contributions

GD was involved in the study concept and design, acquisition of data, analysis and interpretation of data, and drafting of the manuscript. GC was involved in the acquisition of data, analysis and interpretation of data and drafting of the manuscript. JMM critically revised the manuscript for important intellectual content. GG was involved in the acquisition of data and critical revision of the manuscript for important intellectual content. EH was involved in the acquisition of data and critical revision of the manuscript for important intellectual content. GCE was involved in the study concept and design, acquisition of data, critical revision of the manuscript for important intellectual content and study supervision. SVR was involved in the study concept and design, acquisition of data, analysis and interpretation of data, critical revision of the manuscript for important intellectual content and study supervision. All authors read and approved the final manuscript.

## Pre-publication history

The pre-publication history for this paper can be accessed here:

http://www.biomedcentral.com/1741-7015/10/69/prepub
